# Prenatal Developmental Toxicity of ARVIDEX® Herbal Medicine in Albino Wistar Rats

**DOI:** 10.7759/cureus.103279

**Published:** 2026-02-09

**Authors:** Patrick Engeu Ogwang, Kiprotich Joshua, Philbert Katende, Theola R Arisinga, Sarah Kyomugisha, Timothy Neeza, Swase Dominic Terkimbi, Tadele Mekuriya Yadesa, Daniel Chans Mwandah

**Affiliations:** 1 Pharmacy, Mbarara University of Science and Technology, Mbarara, UGA; 2 Pharmacy, Kampala International University, Ishaka-Bushenyi, UGA; 3 Pharmacology and Toxicology, Kampala International University, Ishaka-Bushenyi, UGA; 4 Biochemistry, Kampala International University, Ishaka-Bushenyi, UGA; 5 Pharmacology and Therapeutics, Mbarara University of Science and Technology, Mbarara, UGA

**Keywords:** arvidex®, fetal development, maternal toxicity, polyherbal formulation, pregnancy outcomes, prenatal toxicity, wistar rats

## Abstract

Background

ARVIDEX® is a polyherbal formulation under experimental development for potential immunomodulatory and antiviral applications. Despite preliminary pharmacological promise, evidence regarding its safety during pregnancy is lacking. This study therefore evaluated the prenatal developmental toxicity of ARVIDEX® in pregnant albino Wistar rats.

Methods

Fifty pregnant Wistar rats were randomly allocated to five groups (n=10 per group) and administered ARVIDEX® orally at doses of 250, 500, or 1000 mg/kg, a human equivalent dose (296.7 mg/kg), or distilled water (control) from gestation day 5 to day 19. Maternal endpoints included body weight variation, feed intake, organ weights, and serum biochemical indices. Caesarean section was performed on gestation day 20, followed by assessment of fetal implantation sites, resorption frequency, number of live fetuses, fetal body weight, crown rump length, and external anomalies. Statistical analysis employed one-way analysis of variance (ANOVA) with Dunnett’s or Tukey’s post hoc tests where appropriate. Categorical variables were analyzed using the Chi square or Fisher’s exact test, with statistical significance set at p<0.05.

Results

The administration of ARVIDEX® produced dose-related maternal and developmental toxicity. Significant reductions in maternal weight gain and food intake were observed at doses of 500 and 1000 mg/kg (p<0.05). These doses were also associated with increased relative liver and kidney weights, indicating possible hepatorenal stress. Fetal assessments demonstrated significant decreases in implantation sites, litter size, crown rump length, and placental weight at higher dose levels. An increase in resorption rates and a reduction in both male and female fetuses were also recorded compared with controls.

Conclusion

The findings indicate that ARVIDEX® induces dose-dependent adverse maternal and developmental effects at moderate to high doses, while no statistically significant adverse effects were observed for the measured maternal and fetal endpoints at the human equivalent dose. These data underscore the need for further safety evaluation and support caution against the use of ARVIDEX® above the recommended doses during pregnancy until additional evidence becomes available*.*

## Introduction

Herbal medicines have been utilized for centuries in the management of diverse disease conditions and continue to play a prominent role in primary healthcare systems, particularly in low- and middle-income countries. Their widespread use is largely attributed to affordability, accessibility, and long-standing cultural acceptance. In recent years, there has been renewed global interest in complementary and traditional medicines, including in industrialized settings, reflecting increasing demand for natural therapeutic alternatives alongside conventional pharmaceuticals [1]. A common misconception is that herbal medicines are inherently safe because they are natural. This belief often leads to unsupervised use, including during pregnancy, potentially increasing the risk of adverse maternal and fetal outcomes.

ARVIDEX® is a newly developed polyherbal formulation produced by Jena Herbals Limited (Kampala, Uganda) and derived from the foundational compositions of COVIDEX® and Artemune®. The formulation contains extracts from *Zanthoxylum gilletii*, *Warburgia ugandensis*, *Artemisia annua*, *Moringa oleifera*, and *Persea americana*. Although ARVIDEX® remains in the preclinical development phase, it is being explored for potential use in the management of systemic retroviral infections, based on reported antiviral and immunomodulatory properties of its constituent plants [2,3].

Retroviruses, members of the Retroviridae family, possess an RNA genome and the enzyme reverse transcriptase, which enables the transcription of viral RNA into DNA, an essential step for integration into the host genome. This unique replication mechanism contributes to the pathogenesis of several retrovirus-induced diseases. Human Immunodeficiency Virus (HIV), the causative agent of acquired immunodeficiency syndrome (AIDS), continues to be the most significant retroviral infection worldwide. Other notable retroviruses include human T-lymphotropic virus type 1 (HTLV-1), known to cause adult T-cell leukemia/lymphoma, and HTLV-2, linked to neurological disorders [4].

The AIDS epidemic has persisted as a major global health concern for over four decades, resulting in more than 40 million deaths by 2023 [5]. By 2018, an estimated 25.7 million individuals were living with HIV worldwide, and more than 1.1 million new infections were reported during that year. The African continent remains the most heavily impacted region, accounting for nearly two-thirds of global cases [6]. East and Southern Africa have the highest prevalence, with over 20 million people living with HIV. In Uganda, a country of 41.5 million people in 2018, 1,915,533 individuals were living with HIV/AIDS. That year, there were approximately 53,000 new infections and 23,000 HIV-related deaths. By 2021, 89% of people living with HIV in Uganda were aware of their status, over 92% of those had initiated antiretroviral therapy (ART), and 95% of those on ART were virally suppressed [7]. AIDS is marked by severe immunosuppression, malignancies, wasting, and central nervous system degeneration, among other complications [8].

The World Health Organization currently endorses antiretroviral therapy (ART) as the cornerstone of HIV management, consisting of combination drug regimens designed to suppress viral replication to undetectable levels and thereby reduce the risk of transmission. Although ART substantially improves both longevity and quality of life, it is not curative and is associated with significant side effects. These may include hepatotoxicity, nephrotoxicity, lipid abnormalities, anemia, peripheral neuropathy, hyperglycemia, central nervous system symptoms, headache, insomnia, and cardiovascular complications as well as several perinatal adverse effects [9-11].

Given the limitations of existing ART such as adverse effects, drug resistance, and limited accessibility in low-resource settings, there is a compelling need for alternative or complementary therapeutic strategies. In regions with high HIV prevalence, tuberculosis remains a common co-morbidity, further complicating therapeutic decision making and increasing reliance on adjunctive therapies. In Uganda, herbal medicines are frequently used due to their availability and widespread promotion. As such, polyherbal formulations like ARVIDEX® represent a promising avenue for therapeutic development and require rigorous scientific validation to assess their safety and efficacy.

Given that ARVIDEX® has not yet received regulatory approval and differs from its predecessor products, a dedicated safety evaluation is required. The present study therefore aimed to investigate its potential prenatal developmental toxicity in a rat model, with particular relevance to maternal and fetal safety during pregnancy.

## Materials and methods

Experimental animals

Nulliparous, healthy female Wistar albino rats aged 10-12 weeks and weighing 220-240 g were used in this study. The animals were procured from the Pharmacology Animal House of Kampala International University. They were acclimatized to the laboratory environment for seven consecutive days prior to the experiment. During this period and throughout the study, rats were housed in clean, well-ventilated cages under standard environmental conditions: room temperature of 23±3°C, relative humidity of 50±10%, and a 12-hour light/dark cycle. All animals had access to a standard laboratory diet and clean drinking water ad libitum.

All animal handling and experimental procedures were performed in compliance with internationally recognized standards for the care and use of laboratory animals. Ethical approval was obtained from the Institutional Animal Care and Use Committee of Kampala International University (Approval No: KIU-2024-373). Throughout the study, measures were implemented to minimize animal distress, and the number of animals used was limited to that required for statistical validity, in line with the principles of reduction, refinement, and replacement.

Experimental design

Prenatal developmental toxicity was assessed following Organisation for Economic Co-operation and Development (OECD) Test Guideline No. 414 [12]. For breeding purposes, two female rats were housed with a proven fertile male during the morning for a period of two to four hours. Successful mating was confirmed by microscopic detection of spermatozoa in vaginal smears, and the day of confirmation was designated as gestation day 0 (GD 0), as recommended [13].

Pregnant rats were randomly allocated to five experimental groups (n=10 per group). Group I received a low dose of ARVIDEX® (250 mg/kg), Group II a mid-dose (500 mg/kg), Group III a high dose (1000 mg/kg), Group IV the human equivalent dose (296.7 mg/kg), and Group V served as the control and was administered distilled water at a volume not exceeding 1 mL per 100 g body weight. The human equivalent dose (HEQ) represents a rat equivalent dose calculated from an assumed human daily dose using body surface area normalization. Dose conversion was performed according to the method described by Reagan Shaw et al., using the formula:

$\text{Animal dose (mg/kg)}=\text{Human dose (mg/kg)} \times\left( \frac{Km^{\mathrm{human}}}{Km^{\mathrm{rat}}} \right)$

with Km values of 37 for humans and 6 for rats. Based on this conversion, a dose of 296.7 mg/kg was selected [14]. Maternal and fetal toxicity parameters were assessed throughout the experimental period.

Determination of the effects of ARVIDEX® on prenatal development in Wistar rats

Maternal In-Life Observations and Measurements

Pregnant rats were monitored twice daily for overall health status, survival, and clinical signs of toxicity, including abortion, vaginal bleeding, premature parturition, convulsions, and behavioral abnormalities. Maternal body weights were measured on gestation day 0 before dosing, on day 5 at the initiation of treatment, and subsequently at three day intervals until gestation day 20. Food intake was recorded on gestation days 0 and 5 and then at three day intervals throughout the dosing period to assess nutritional consumption [13].

On gestation day 20, rats were anesthetized via intraperitoneal injection of pentobarbital sodium (150 mg/kg body weight), as previously described, and humanely euthanized for necropsy [15].

Maternal Biochemical and Hematological Evaluation

Following euthanasia, approximately 3-5 mL of blood was collected from each dam by cardiac puncture and transferred into plain and heparinized vacutainer tubes. After allowing the samples to clot at room temperature for one hour, they were centrifuged at 3,000 rpm for 10 minutes. Serum was analyzed using an Automated Clinical Chemistry Analyzer (HumaStar 80, Human Diagnostics, Germany) for biomarkers of hepatic and renal function: alanine aminotransferase (ALT), alkaline phosphatase (ALP), aspartate aminotransferase (AST), blood urea nitrogen (BUN), and creatinine [13].

Hematological parameters were assessed from heparinized blood samples using standard techniques [16]. The parameters evaluated included total white blood cell (WBC) count, WBC differentials (neutrophils, lymphocytes, monocytes, eosinophils, and basophils), red blood cell (RBC) count, hemoglobin concentration (Hb), hematocrit (HCT), mean corpuscular volume (MCV), mean corpuscular hemoglobin (MCH), mean corpuscular hemoglobin concentration (MCHC), and platelet count (PCH). All analyses were performed using an automated hematology analyzer (Genrui KT-6610; Genrui Biotech, Shenzhen, China) at the Clinical Chemistry Laboratory of Kampala International University Teaching Hospital.

Maternal Post-Mortem Examination

After blood collection, the dams were euthanized using pentobarbital intraperitoneally 60 mg/kg and subjected to macroscopic examination. The liver, kidneys, ovaries, placentae, and gravid uterus were excised, cleaned of adherent tissues, and weighed.

Corpora lutea in both ovaries were counted using a dissecting microscope (XTL3101, GX Optical, Haverhill, UK). The gravid uterus was opened longitudinally to expose the fetuses after removal of the chorionic and amniotic membranes. The placentae were then examined and weighed.

The number of implantation sites, live and dead fetuses, and early/late resorptions were recorded. Live fetuses were weighed, their sex identified as male or female and measured for crown-rump length. They were examined for external abnormalities of the craniofacial region, trunk, genitalia, limbs, and tail. Fetal assessments were limited to external morphological examination. Detailed skeletal or visceral examinations were not performed.

Procurement of ARVIDEX®

ARVIDEX® was obtained directly from Jena Herbals Ltd., Luzira, Kampala. Upon receipt, the product underwent preliminary verification to confirm authenticity through inspection of labeling, batch number, packaging integrity, and physical attributes, ensuring it was uncompromised prior to use.

Statistical analysis

The litter was considered the experimental unit for analysis of reproductive and fetal outcomes to account for non independence of fetuses within the same dam. Quantitative data were expressed as mean±SEM. Group comparisons were performed using one-way analysis of variance (ANOVA), followed by Tukey’s or Dunnett’s post hoc tests as appropriate. Maternal and fetal continuous variables were analyzed using ANOVA-based methods, while categorical outcomes were evaluated using the chi-square test or Fisher’s exact test. Statistical significance was defined as p<0.05.

Quality control

To ensure the reliability and reproducibility of the study results, stringent quality control measures were implemented throughout the experimental period. These included the appropriate transportation and storage of ARVIDEX® under manufacturer-recommended conditions to maintain product integrity. All experimental data were recorded daily in a structured and secure manner, with each entry verified to prevent duplication or omission.

Only analytical-grade chemicals and reagents were utilized in all procedures to maintain experimental accuracy. Non-parous, inbred female Wistar rats were selected for the study due to their increased sensitivity in toxicity assessments. Baseline physiological and hematological parameters were established prior to dosing to allow comparative analysis post-treatment. All histopathological assessments were carried out by a licensed pathologist to ensure diagnostic accuracy.

## Results

Effects of ARVIDEX® on maternal outcomes in pregnant Wistar rats

Maternal Weight and Food Intake

A significant reduction in maternal weight was observed in rats treated with 500 mg/kg (p=0.0005) and 1000 mg/kg (p<0.0001) of ARVIDEX® compared to the control group (Table 1).

**Table 1:** Effects of ARVIDEX® on body weight changes in Pregnant experimental rats Values are expressed as mean ± SEM. *p<0.05 compared with normal control, one way ANOVA followed by Tukey’s post hoc test. HEQ, human equivalent dose.

Treatment group	Initial weight (g)	Final weight (g)	Weight gain (%)
Normal control	210.5±12.3	245.2±14.1	16.5±2.1
Arvidex® 250 mg/kg	208.7±11.9	238.1±13.5	14.1±1.8
Arvidex® 500 mg/kg	212.4±13.5	225.6±12.8	6.2±1.5
Arvidex® 1000 mg/kg	209.8±14.2	198.7±11.9	-5.3±1.2
Arvidex® 296.7 mg/kg (HEQ)	207.3±12.8	232.4±13.1	12.1±1.9
ANOVA F (df)	0.022 (4, 45)	1.87 (4, 45)	25.4 (4, 45)
P value	0.9990	0.1329	<0.0001*

**Figure 1:**
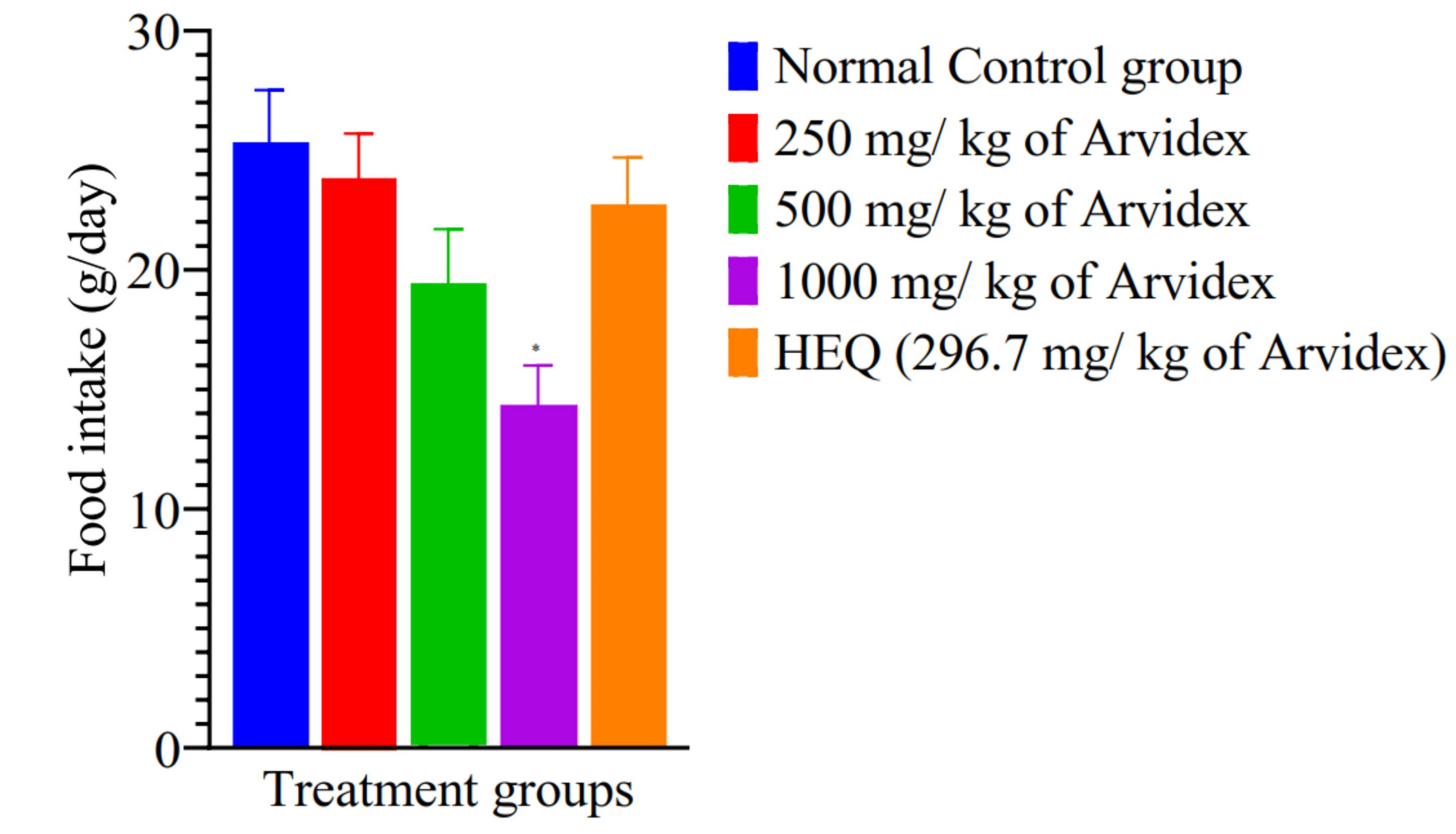
Food intake across groups in pregnant female wistar rats treated with ARVIDEX® herbal extract HEQ: Human equivalent dose, mg/kg: milligrams per kilogram bodyweight

A dose-dependent decline in food intake was also noted, with the lowest intake recorded in the 1000 mg/kg group. This reduction was statistically significant (p=0.0013) compared to the control group (Figure 1).

Organ Weights and Relative Organ Weights

While absolute liver, kidney, and spleen weights showed no significant differences between groups, relative liver weight (p=0.0179) and relative spleen weight (p=0.0267) were significantly increased in the 1000 mg/kg group (Table 2).

**Table 2:** Effects of ARVIDEX® on relative and absolute organ weights in pregnant rats Values are expressed as mean±SEM. *p<0.05 compared with normal control, one-way ANOVA followed by Tukey’s post hoc test. BW, body weight; HEQ, human equivalent dose; SEM: standard error of the mean; ANOVA: analysis of variance.

Organ	Normal control	Arvidex® 250 mg/kg	Arvidex® 500 mg/kg	Arvidex® 1000 mg/kg	Arvidex® 296.7 mg/kg (HEQ)	ANOVA F (df)	P value
Liver (g)	8.2±0.9	8.5±1.1	9.8±1.3	11.4±1.5	8.7±1.0	1.23 (4, 45)	0.3102
Liver/BW (%)	3.3±0.4	3.6±0.5	4.4±0.6	5.7±0.8	3.7±0.5	2.78 (4, 45)	0.0379 *
Kidney (g)	1.5±0.2	1.6±0.3	1.8±0.3	2.1±0.4	1.6±0.2	0.679 (4, 45)	0.6104
Kidney/BW (%)	0.6±0.1	0.7±0.1	0.8±0.1	1.1±0.2	0.7±0.1	2.313 (4, 45)	0.0721
Spleen (g)	0.4±0.05	0.4±0.06	0.5±0.07	0.6±0.08	0.4±0.05	2.0 (4, 45)	0.1092
Spleen/BW (%)	0.16±0.02	0.17±0.03	0.22±0.04	0.30±0.05	0.17±0.03	2.722 (4, 45)	0.0411 *

Effects of ARVIDEX® on Pregnancy Outcomes

ARVIDEX® administration resulted in significant alterations in key pregnancy outcomes among the treated pregnant Wistar rats. The findings revealed a dose-dependent negative effect on implantation, fetal viability, and litter size.

Rats treated with 1000 mg/kg demonstrated a statistically significant reduction in the number of implantation sites compared to the normal control group (p=0.0289; Table 3). Moreover, the incidence of fetal resorption was significantly elevated in groups treated with 500 mg/kg and 1000 mg/kg of the extract (p=0.0191 and p=0.0032, respectively), suggesting potential embryotoxic effects at higher doses.

A notable decline in the number of live fetuses was observed in rats treated with both 500 mg/kg (p=0.0376) and 1000 mg/kg (p<0.0001) of the extract, in contrast to the control group. This indicates a dose-related adverse effect on fetal survival.

Furthermore, a significant decrease in the number of both male and female fetuses was recorded in the treatment groups receiving 500 mg/kg (male: p=0.0416; female: p=0.0208) and 1000 mg/kg (male and female: p<0.0001) of ARVIDEX® when compared to the control group (Table 3).

**Table 3:** Effects of ARVIDEX® on reproductive parameters in pregnant Wistar rats Values are expressed as mean±SEM. *p<0.05 compared with normal control, one-way ANOVA followed by Tukey’s post hoc test. HEQ, human equivalent dose. Dead fetuses showed no variance across groups; therefore, statistical analysis was not applicable. SEM: Standard error of the mean; ANOVA: analysis of variance.

Parameter	Normal control	Arvidex® 250 mg/kg	Arvidex® 500 mg/kg	Arvidex® 1000 mg/kg	HEQ 296.7 mg/kg	ANOVA F (df)	P value
Number of litters	10	10	10	8	10	-	-
Implantation sites	12.3±1.5	11.8±1.2	10.1±1.4	7.2±1.1	11.5±1.3	2.478 (4, 45)	0.0574
Resorptions	1.1±0.3	1.5±0.4	2.9±0.5	3.3±0.6	1.3±q0.3	5.326 (4, 45)	0.0013 *
Live fetuses	11.4±1.3	10.2±1.1	7.3±1.0	3.9±0.8	10.8±1.2	8.141 (4, 45)	<0.0001*
Dead fetuses	0.0±0.0	0.0±0.0	0.0±0.0	0.0±0.0	0.0±0.0	-	-
Male fetuses	5.8±0.7	5.2±0.6	3.7±0.5	2.0±0.4	5.5±0.6	7.756 (4, 45)	<0.0001*
Female fetuses	5.6±0.6	5.0±0.5	3.6±0.5	1.9±0.3	5.3±0.5	9.821 (4, 45)	<0.0001*

Effects of ARVIDEX® on Fetal Growth Parameters

Fetal outcomes showed significant reductions in litter weight (p=0.0039), crown-rump length (p=0.0222), and placental weight (p=0.0155) in the 1000 mg/kg group, indicating impaired fetal growth (Table 4).

**Table 4:** Effects of ARVIDEX® on fetal growth and placental parameters in pregnant rats Values are expressed as mean±SEM. *p<0.05 compared with normal control, one-way analysis of variance (ANOVA) followed by Tukey’s post hoc test. HEQ, human equivalent dose.

Parameter	Normal control	Arvidex® 250 mg/kg	Arvidex® 500 mg/kg	Arvidex® 1000 mg/kg	HEQ 296.7 mg/kg	ANOVA F (df)	P value
Litter weight (g)	3.8±0.4	3.5±0.3	2.9±0.3	2.1±0.3	3.6± 0.4	4.042 (4, 45)	0.0070*
Crown-rump length (mm)	34.2±2.1	32.5±1.9	29.8±1.7	26.7±1.5	33.1±2.0	2.658 (4, 45)	0.0448*
Placental weight (g)	0.52±0.06	0.49±0.05	0.41±0.04	0.32±0.03	0.50±0.05	3.095 (4, 45)	0.0247*

## Discussion

This study investigated the potential prenatal developmental toxicity of ARVIDEX® in pregnant albino Wistar rats and demonstrated clear dose-dependent adverse effects on maternal health and fetal development. The findings are consistent with published evidence indicating that certain polyherbal formulations may exert toxic effects when administered at elevated doses. The discussion interprets these findings in relation to the pharmacological and ethnobotanical properties of the individual plant components of ARVIDEX®, as described in the literature review.

Effects on maternal weight and food intake

The reduction in maternal body weight gain observed at 500 and 1000 mg/kg suggests systemic maternal toxicity. This effect is supported by the concurrent decline in food intake, which is commonly recognized as an early indicator of adverse physiological response to toxic exposure [17]. These findings are consistent with prior reports that documented weight loss in pregnant rats administered high doses of *A. annua* extract [18].

Animals treated with the 250 mg/kg and human equivalent (HEQ) doses of ARVIDEX® did not demonstrate significant alterations in weight gain or food consumption, indicating a potential safety margin at lower doses. This is consistent with previous findings, which reported no adverse maternal effects at low doses of *M. oleifera*, whereas higher doses resulted in toxic manifestations [19].

Organ weights and maternal toxicity

At the highest dose, increases in relative liver and spleen weights were observed without corresponding changes in absolute organ mass. This pattern likely reflects reduced overall body weight rather than true organ hypertrophy [20]. However, physiological stress on metabolically active organs cannot be ruled out and may still be linked to oxidative stress, impaired excretion, or phytochemical burden [21]. The absence of absolute weight changes implies a slow, progressive toxicity rather than acute damage.

Pregnancy outcomes

Significant reductions in implantation sites and increased fetal resorptions at higher doses indicate embryotoxic potential. Such outcomes have been previously associated with exposure to high concentrations of certain phytochemicals, including artemisinin derivatives [22]. Increased fetal resorption rates are often indicative of embryotoxicity and may be mediated by mechanisms such as oxidative stress, inflammatory responses, or endocrine disruption, reduced nutrient supply due to reduced food intake and reduced body weights, all of which have been linked to high-dose exposure to certain herbal constituents [23].

The fact that no significant fetal resorption was observed at the HEQ dose implies a threshold below which ARVIDEX® may be safer for use during pregnancy. However, the observed dose-dependent effects underscore the importance of regulating herbal medicines during pregnancy, especially those with multiple bioactive constituents [18]. These results align with the cautionary stance recommended by the National Drug Authority on the unregulated use of herbal products in pregnant populations [24].

Fetal outcomes

The significant reductions in litter weight, crown-rump length, and placental weight at the 1000 mg/kg dose indicate that ARVIDEX® may have teratogenic potential at higher doses. The observed reductions in these key fetal growth parameters are consistent with findings from previous studies investigating other herbal products, such as *A. annua* and *W. ugandensis*, both of which were associated with adverse fetal development in animal models [17,25].

The reduction in placental weight, in particular, is noteworthy, as it suggests impaired placental function. The placenta plays a critical role in nutrient and oxygen transfer to the fetus, and a decrease in placental mass could compromise fetal growth and survival [26].

Possible mechanisms of toxicity

The toxicity observed with high doses of ARVIDEX® administration is likely mediated by multiple, interrelated mechanisms.

One key pathway may involve oxidative stress, particularly linked to certain alkaloids present in the extract. Alkaloids have been reported to induce the generation of reactive oxygen species (ROS), which can disrupt cellular function through lipid peroxidation, DNA damage, and protein oxidation, ultimately contributing to maternal toxicity and fetal impairment [27].

Another plausible mechanism is hormonal disruption. *A. annua*, a component of ARVIDEX®, has been documented to alter the balance of estrogen and progesterone, hormones essential for successful implantation, embryogenesis, and placental function. Disruption in these hormonal levels could therefore impair the establishment and maintenance of pregnancy [17].

Additionally, direct embryotoxicity may contribute to the adverse outcomes. Certain sesquiterpenes from *W. ugandensis* and alkaloids from *Z. gilletii* have demonstrated cytotoxic activity, which may interfere with normal mitotic processes or induce apoptosis in rapidly dividing embryonic cells. Such effects can lead to embryonic resorption, fetal growth retardation, or structural malformations [28]. Nutrient deficiency following placental malfunction observed may also have contributed to fetal resorption.

Together, these mechanisms likely operate synergistically to produce the maternal and fetal effects observed in this study.

Implications for human health

The results of this study underscore the potential teratogenic risks of ARVIDEX® at doses higher than HEQ doses, particularly during pregnancy. While no significant toxicity was observed at the HEQ dose, the dose-dependent effects observed in the higher dose groups suggest that excessive use of ARVIDEX® could pose significant risks to both maternal and fetal health. These findings support the importance of preclinical safety evaluations for herbal medicines before they are used in vulnerable populations, such as pregnant women [24].

Study limitations

Although the study followed established guidelines for prenatal developmental toxicity assessment, several limitations should be acknowledged. The work used only one animal species and strain, therefore species-specific differences in metabolism and susceptibility cannot be excluded. The study evaluated gross fetal outcomes but did not include detailed skeletal or visceral examinations that might identify subtle developmental effects. Biochemical analysis was limited to maternal tissues, so mechanistic pathways underlying fetal toxicity remain speculative. The dosing period covered organogenesis; however, repeated dose studies across the entire gestational period would offer more comprehensive reproductive safety data. Furthermore, the study evaluated the prenatal toxicity of the finished polyherbal formulation. Individual constituent herbs, specific phytochemicals, neurotoxicity endpoints, antiviral efficacy, and paternal exposure effects were not assessed. These represent important areas for future investigation. These limitations indicate that the findings should be interpreted with caution and highlight the need for additional studies, including mechanistic assays and multi species evaluations.

## Conclusions

This study suggests that ARVIDEX® produces dose-dependent prenatal developmental toxicity in pregnant albino Wistar rats. Moderate to high doses were associated with adverse maternal outcomes, impaired pregnancy parameters, and restricted fetal growth, whereas the human equivalent dose did not elicit detectable toxicity.

These findings highlight the importance of rigorous preclinical safety assessment and support cautious use of herbal formulations during pregnancy. Future research should focus on elucidating the histopathological and molecular mechanisms of toxicity and assessing the long-term impact of prenatal ARVIDEX® exposure to further refine its safety profile.
